# Circulation of a novel strain of dolphin morbillivirus (DMV) in stranded cetaceans in the Mediterranean Sea

**DOI:** 10.1038/s41598-019-46096-w

**Published:** 2019-07-05

**Authors:** Francesco Mira, Consuelo Rubio-Guerri, Giuseppa Purpari, Roberto Puleio, Giulia Caracappa, Francesca Gucciardi, Laura Russotto, Guido Ruggero Loria, Annalisa Guercio

**Affiliations:** 1 Istituto Zooprofilattico Sperimentale della Sicilia “A. Mirri”, Palermo, 90129 Italy; 2 Fundación Oceanografic de la Comunitat Valenciana, Valencia, 46013 Spain; 3 VISAVET-Animal Health Department, Veterinary School, Complutense University, Madrid, 28040 Spain

**Keywords:** RNA sequencing, Virology

## Abstract

Dolphin morbillivirus (DMV) has been responsible for several outbreaks of systemic infection and has resulted in cetacean strandings in the Mediterranean. In August-October 2016, seven striped dolphins (*Stenella coeruleoalba*) stranded on the Sicilian coastline (Italy) tested positive for DMV. Tissue samples from brain, lung, pulmonary lymph nodes, heart, spleen, liver, stomach, intestine, kidneys and urinary bladder, as well as blowhole swabs, were collected during necropsy for molecular diagnostics and pathology studies. Extracted tissue RNA was screened for DMV by real-time reverse transcription polymerase chain reaction (PCR). Some tissues exhibited microscopic lesions that were consistent with DMV infection on histopathological and immunohistochemical grounds. Conventional reverse transcription PCR to target partial nucleoprotein and phosphoprotein genes yielded sequences used to genetically characterize the associated DMV strain. DMV RNA was detected by both PCR assays in all tested tissues of the seven dolphins, which suggests systemic infections, but was absent from another dolphin stranded on the Sicilian coastline during the same period. The partial phosphoprotein and nucleoprotein gene sequences from the positive dolphins were 99.7% and 99.5% identical, respectively, to the DMV sequences recently observed in cetaceans stranded on the Spanish Mediterranean. Our study suggests that this DMV strain is circulating in the Mediterranean.

## Introduction

*Cetacean morbillivirus* (CeMV) is an enveloped negative-sense RNA virus classified in the genus *Morbillivirus* in the family *Paramyxoviridae*^1^. Classically, three main CeMV clades have been described: dolphin morbillivirus (DMV)^2^ and porpoise morbillivirus (PMV)^1^, which have caused massive mortalities, and pilot whale morbillivirus (PWMV)^3,4^, described in only two specimens. After the identification of three additional CeMV-related strains in cetaceans from Hawaii, Brazil and Australia^5–7^, the different variants have been clustered according to divergences in phosphoprotein (P) gene sequences into two CeMV lineages. The CeMV-1 lineage includes DMV, PMV, PWMV and the more recently reported strain from Hawaii named Beaked Whale Morbillivirus (BWMV)^8^. The other lineage, CeMV-2, includes the strains detected in a Guiana dolphin (*Sotalia guianensis*) from Brazil and in an Indo-Pacific bottlenose dolphin (*Tursiops aduncus*) from Australia^8,9^.

In the past 25 years, different CeMV strains have caused pulmonary and neurological diseases in cetaceans that have led to stranding events involving massive numbers of animals to just one cetacean or a few^9,10^. The first recognized CeMV epizootic occurred in 1987–88 on the Atlantic coast of the USA^11^, when approximately 50% of the bottlenose dolphin (*Tursiops truncatus*) population died. An epizootic in the Mediterranean Sea was reported in 1990 after around 1000 Mediterranean striped dolphins (*Stenella coeruleoalba*)^12^ were stranded. A third CeMV epizootic in the Gulf of Mexico led to the death of at least 100 bottlenose dolphins^13,14^. Two more epizootics occurred in the Mediterranean Sea in 2007 and 2011, when more than 200 and 50 striped dolphins died, respectively^15–17^. DMV infections have been reported on the Italian coastline of the Mediterranean Sea in different cetacean species, including fin whale (*Balaenoptera physalus*), sperm whale (*Physeter macrocephalus*), Cuvier’s beaked whale (*Ziphius cavirostris)*, striped dolphin and bottlenose dolphin^18–24^. This suggests that DMV remains a major threat to cetaceans. From 2012 to 2017, a novel DMV strain of Atlantic origin has been repeatedly identified in striped dolphins stranded on the Spanish Mediterranean coastline^25^.

We herein describe seven DMV infection cases in striped dolphins that stranded on Italian coastlines of Sicily, in the Mediterranean and Ionian Seas, in 3 months of 2016. Partial sequences of the nucleoprotein (N) and P genes of DMV proved identical in these seven animals and were closest to those of recent Spanish Mediterranean isolates. A comparison of these sequences with those of other reported CeMV strains (including a DMV isolated from a fin whale stranded in 2013 on the Italian coast^22^ that was sequenced^26^) was made to better understand the genetic relationships and molecular epidemiology of DMV in the Mediterranean Sea.

## Results

**Figure 1:**
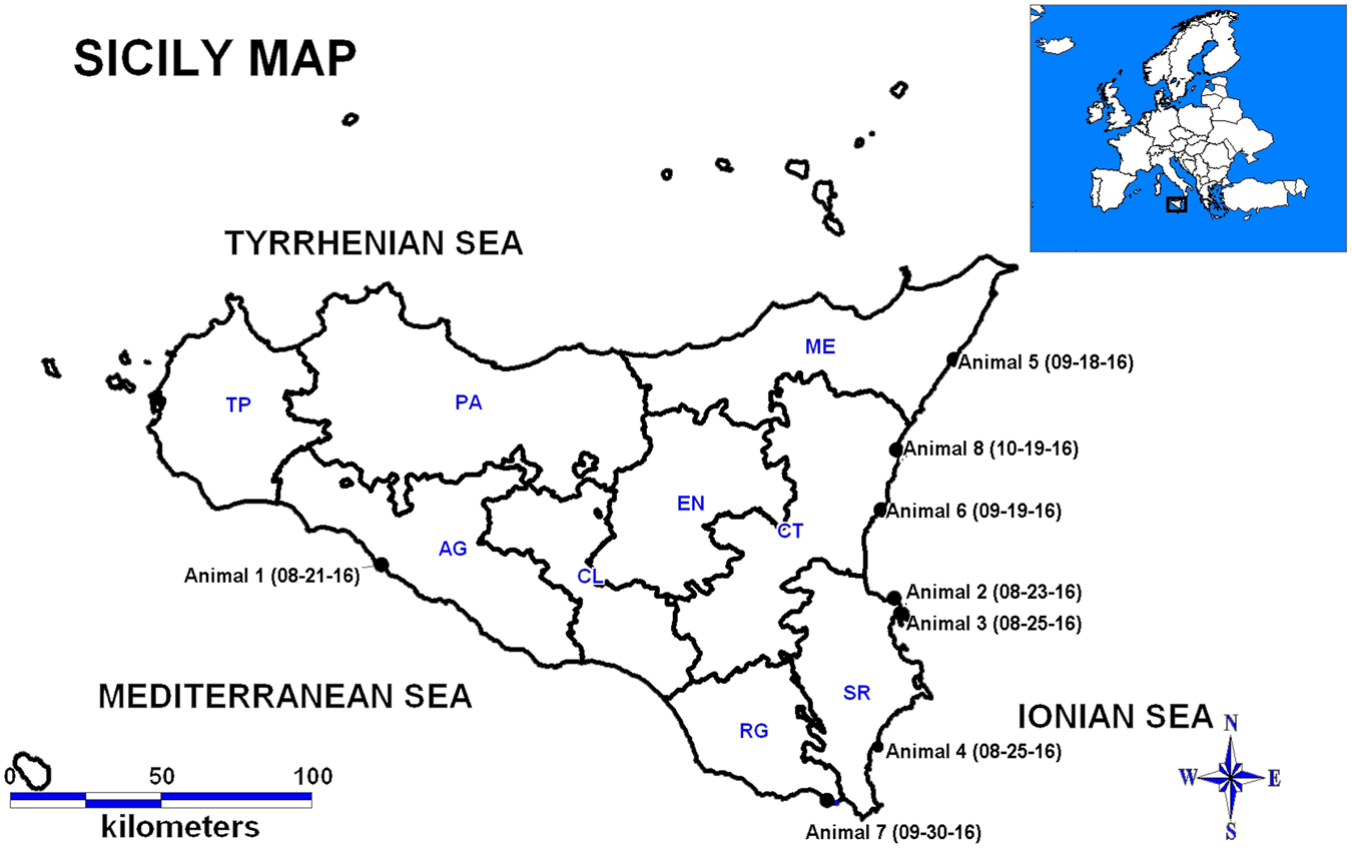
Stranding locations of the eight striped dolphins (*Stenella coeruleoalba*) that were stranded on the Sicilian coast in 2016. Black dots mark the location of strandings. The stranding date (month-day-year) is indicated next to each animal’s identification number. The map was created by the MapInfo Professional v8.5 software.

Eight striped dolphins, stranded between August and October 2016 on the coastline of Sicily, were studied (Fig. 1 and Table 1). Animals 1, 2, 4 and 7 were freshly dead (code 2, grading according to^27^), animals 3, 6 and 8 displayed moderate autolysis (code 3), and animal 5 exhibited a more advanced level of autolysis (code 4). On external examination, the animals appeared normal and showed no signs of starvation/wasting. At necropsy, all the animals had empty stomachs. No significant macroscopic lesions were identified, although advanced autolysis in animal 5 precluded a clear-cut assessment of internal macroscopic lesions (Supplementary Table S1).

**Table 1:** Stranding date, stranding location, gender and size of the eight striped dolphins (*Stenella coeruleoalba*) stranded on the Sicilian coastline described herein.

Animal	IZS ID*	Specie	Stranding date	Stranding location	Gender	Size (cm)	Age
**1**	PA30087/2016	*Stenella coreuleoalba*	21-Aug-2016	Eraclea Minoa (AG) (37°23′39′′N, 13°17′28′′E)	Female	169	Preadult
**2**	PA30237/2016	*Stenella coreuleoalba*	23-Aug-2016	Augusta (SR) (37°15′0.2′′N, 15°13′18′′E)	Female	207	Adult
**3**	PA30619/2016	*Stenella coreuleoalba*	25-Aug-2016	Augusta (SR) (37°15′0.2′′N, 15°13′18′′E)	Female	133	Juvenile
**4**	PA30356/2016	*Stenella coreuleoalba*	25-Aug-2016	Noto (SR) (36°52′11.2′′N, 15° 08′ 14.4′′ E)	Male	98	Calf
**5**	PA33448/2016	*Stenella coreuleoalba*	18-Sep-2016	Messina, loc. Tremestieri (ME) (38°8′9,2′′N, 15°31′46′′E)	Undetermined	111	Calf
**6**	PA33442/2016	*Stenella coreuleoalba*	19-Sep-2016	Riposto (CT) (37°43′55′′N, 15°12′19′′E)	Female	192	Adult
**7**	PA35875/2016	*Stenella coreuleoalba*	30-Sep-2016	Ispica (RG) (36°41′36′′N, 14°58′25′′E)	Female	205	Adult
**8**	PA39573/2016	*Stenella coreuleoalba*	19-Oct-2016	Giardini Naxos (ME) (37°50′9′′N, 15°16′22′′E)	Undetermined	190	Adult

*IZS ID: Istituto Zooprofilattico della Sicilia identification code.

**Figure 2:**
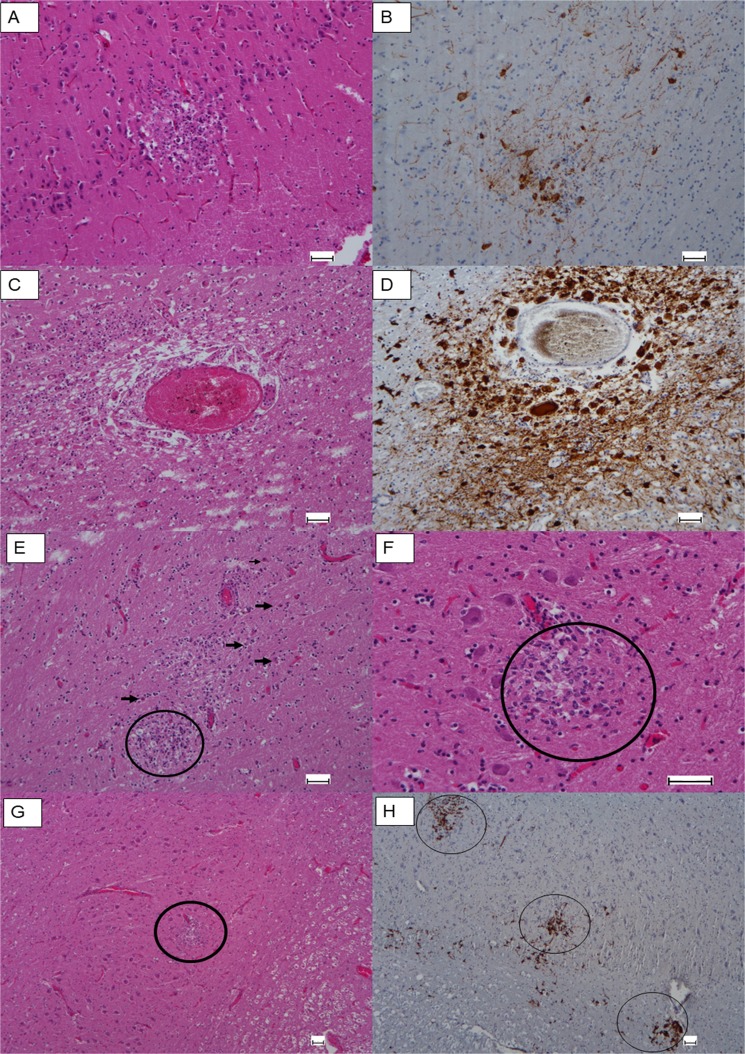
Cerebral microscopic lesions and results of immunohistochemistry against dolphin morbillivirus (DMV) in the striped dolphins (*Stenella coeruleoalba*) stranded in Sicily coast, Italy, in 2016. (**A**) Animal 1: Multifocal gliosis in the frontex cortex. H&E. Scale bar: 50 µm. (**B**) Animal 1: Serial slide of area shown in A. Massive presence of DMV antigen in neuronal bodies and punctate staining in the neutrophile. DMV immunostaining. Scale bar: 50 µm. (**C**) Animal 1: Perivascular infiltration of leukocytes, gemistocytes and syncytial cells in cerebral cortex. H&E. Scale bar: 50 µm. (**D**) Animal 1: serial slide of the area shown in C. Presence of DMV antigen in cerebral cortex perivascular area. DMV Immunostaining. Scale bar: 50 µm. (**E**) Animal 2: Glial proliferation (circle) and gemistocytic astrocytes (arrows) in the parietal cortex. H&E. Scale bar: 50 µm. (**F**) Animal 6: Malacia and glial proliferation (circle) in medulla oblongata. H&E. Scale bar: 50 µm. (**G**) Animal 7: Gliosis and hyperplasia (circle) of blood vessels in the medulla oblongata. H&E. Scale bar: 50 µm. (**H**) Animal 7: serial slide of the area shown in G. Presence of DMV antigen (circles) in the medulla oblongata. DMV immunostaining. Scale bar: 50 µm.

The histological analysis evidenced the severest lesions in the brains of animals 1, 2, 6 and 7, which presented non-suppurative meningoencephalitis with gliosis, neuronal degenerative changes, lymphocytic perivascular cuffing, glial nodules and multinucleate syncytial cells (Fig. 2 and Supplementary Table S1). No other important lesion was found in animals 1, 2, 4, 6 and 7. Animals 3, 5 and 8 presented moderate to advanced autolysis, which did not allow clear microscopic lesion identifications to be made. Except for animals 4 and 8, immunopositivity for the nucleoprotein of canine distemper virus (CDV) was observed in the neurons and gitter cells in the brains of all the animals (Table 2).

**Table 2:** Tissue samples from the striped dolphins (*Stenella coeruleoalba*) that stranded on the Sicilian coastline, Italy, in 2016, tested by Dolphin morbillivirus Universal Probe Library (UPL) real-time reverse transcriptase PCR (DMV UPL RT-PCR) and immunohistochemistry (IHC) for morbilliviral antigen.

Animal	Tissue sampled										
	DMV UPL RT-PCR										IHC
	Brain	Lung	Pulm. LN	Spleen	Kidney	Liver	Intestine	Blow. swab	Ur. bladder	Cerebellum	Brain
1	+°	+°	+	+	+	+	+	+	+	+	+
2	+	+°	+°	+	+	+	+	+	+	+	+
3	+	+	+°	+°	+	+	+		+		+
4	−	−	−	−	−	−	−	−	−	−	−
5	+	+°	+	+°	+	+					
6	+	+°	+	+	+	+			+°		+
7	+	+°	+°	+	+	+		+			+
8	+	+°	+°	+	+	+					_

The tested organs are shown in grey. Positive samples are marked by a (+) and negative ones by an (−). Untested organs are shown in white. °Samples from which fragments of the phosphoprotein (P) and nucleoprotein (N) DMV genes were sequenced using cDNA.

On virological analyses, all the tested tissues, except those of animal 4, were positive for CeMV by real-time PCR (rt PCR), while all eight dolphins tested negative for adenovirus and herpesvirus by conventional PCR assays. All CeMV positive samples (Table 2) were confirmed to be positive by subsequent conventional reverse transcription PCR (RT-PCR) assays run to target genes P and N (amplicons of 429 bp and 287 bp, respectively).

The Sanger sequencing of the partial P gene amplicon of all the positive animals yielded the same sequence. The same observation was made with the partial N gene sequence, which clearly evidenced that the same DMV strain had infected these seven animals.

Compared to the CeMV sequences deposited in the GenBank (http://www.ncbi.nlm.nih.gov/nuccore), our present sequences displayed high nucleotide identities with the sequences obtained from a striped dolphin that stranded in 2012 on the Spanish Mediterranean coast in the Valencian Community (approx. 38°–40°N)^25^ (GenBank accession numbers KC572861 and MG000863 for P and N gene sequences, respectively), with only one synonymous change noted in each examined gene fragment (corresponding to 99.75% and 99.5% identities in the partial P and N gene sequences compared, respectively).

The P gene sequence displayed complete nucleotide identity with the P gene sequence from a striped dolphin stranded in the Canary Islands in 2011^28^ (KJ139454), as well as 98.4–99.75% nucleotide identity with other P gene sequences from striped dolphins stranded in 2012–2014 on the northwestern Atlantic coast of the Iberian Peninsula (KT878661, KT878657, KT878656, KP835987^29^), from fin whales stranded in 2016 in Denmark^30^ (KY681807) and in 2011 and 2013 on the Italian Tyrrhenian Sea coastline (KU977450^22^, KR337460^18^) (Supplementary Table S2).

In turn, the N gene sequence showed 98.5–97.9% nucleotide identity with the sequences obtained from a white-beaked dolphin (*Lagenorhynchus albirostris*) stranded on the Fresian coast of Germany in 2007 (EF469546)^31^, from a Cuvier’s beaked whale (KX237510)^24^, from a fin whale (KU977449)^22^ stranded on the Italian coast between 2011 and 2013, and from a striped dolphin and a long-finned pilot whale stranded on the Mediterranean coast of Spain in 2007 (HQ829973, HQ829972)^32^ (Supplementary Table S3).

The comparison of the P and N gene sequences with the sequences reported in previously stranded cetaceans, revealed in the partial P gene sequence six synonymous and four non-synonymous nucleotide changes (responsible for amino acid substitutions: Gly116Ser, Ser163Asn, Gly249Ser and Arg253Gln), whereas eight synonymous changes were found in the partial N gene sequence (Table 3).

**Table 3:** Synonymous and non-synonymous sequence variations among dolphin morbillivirus (DMV) strains.

Isolate (accession n.) Country, year	Phosphoprotein gene nucleotide (amino acid) positions										Nucleoprotein gene nucleotide positions							
	346 (116)	402	447	488 (163)	618	633	657	745 (249)	758 (253)	760	663	664	717	732	783	811	942	945
DMV_Gme/2007 (HQ829972)† Spain, 2007	G (Gly)	T	C	G (Ser)	A	A	G	G (Gly)	G (Arg)	C	T	C	T	A	C	T	A	T
DMV_Sc/2007 (HQ829973)† Spain, 2007	–	–	–	–	–	–	–	–	–	–	–	–	–	–	–	–	–	–
DMV_Zca/2015 (P: KX237511)† (N: KX237510)† Italy, 2015	–	–	–	–	–	–	–	–	–	–	–	–	–	–	–	–	–	–
DMV_Bph/2013 (KU977450)† (KU977449)† Italy, 2013	A (Ser)	–	–	–	–	–	–	A (Ser)	–	–	–	–	–	–	–	–	–	–
DMV Sc/2016 (P: MG000861) (N: MG000862) Italy, 2016	A (Ser)	C	T	A (Asn)	G	G	A	A (Ser)	A (Gln)	T	C	A	C	T	T	C	T	C
MV Sc/2012 (P: KC572861) (N: MG000863) Spain, 2012	A (Ser)	C	T	A (Asn)	G	G	–	x	x	x	x	x	x	T	T	C	T	C

Nucleotide and deduced amino acid (in brackets) variations in the P and N gene sequences in the DMV strains. Isolates are named with an abbreviation for the scientific name of the species of the infected cetacean (Gme, *Globicephala melas*; Sc, *Stenella coeruleoalba*; Zca, *Ziphius cavirostris*; Bph, *Balaenoptera physalus*) and the year of description. All the genomic positions refer to the reference strain sequences (HQ829972, HQ829973). ^†^Reference strain sequences used for comparisons. ^x^ nucleotide residue not available for this DMV strain.

**Figure 3:**
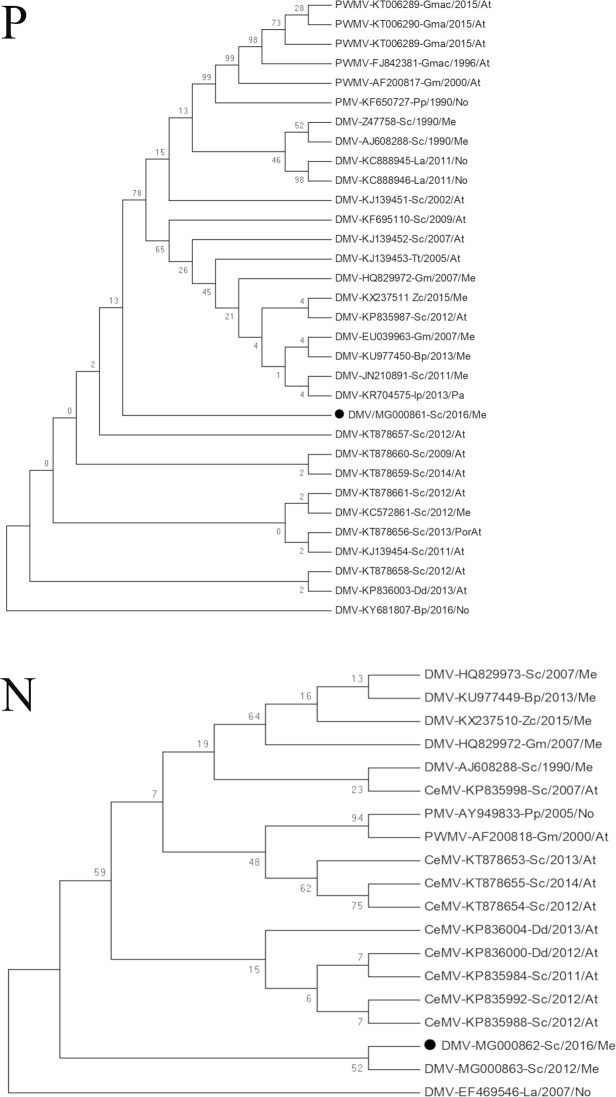
Phylogenetic analysis. Maximum likelihood trees based on a 250-nucleotides (nt 401–650) fragment of DMV phosphoprotein (P) and on a 192-nucleotides (nt 754–945) fragment of DMV nucleoprotein (N) gene sequences. The name of each sequence comprises the virus strain name (DMV, dolphin morbillivirus; PMV, porpoise morbillivirus; PWMV, pilot whale morbillivirus), GenBank accession number, cetacean species host (Sc, *Stenella coeruleoalba*; Gm, *Globicephala melas*; Gma, *Globicephala macrorhynchus;* Tt, *Tursiops truncatus*; Zc, *Ziphius cavirostris*; Bp, *Balaenoptera physalus*; Ip, *Indopacetus pacificus*; La, *Lagenorhynchus albirostris;* Pp, *Phocoena phocoena*, Dd, *Delphinus delphis*), year and geographic area of stranding (At, Atlantic Ocean; Me, Mediterranean Sea; No, North Sea; Pa, Pacific Ocean). Black dots (●) indicate the sequence obtained from one of the seven striped dolphins (*Stenella coeruleoalba*) stranded on the Sicilian coastline, Italy, in 2016.

The phylogenetic trees based on the partial P and N gene sequences support a unique clade that encompasses the present sequences, those of a year 2012 isolate from the Spanish Mediterranean, and Atlantic-found sequences (Fig. 3). A p-distance value close to 0 was observed in the tree for the P gene of these two Mediterranean sequences and for the sequences from the Atlantic stranding events that occurred in northwestern Spain and Portugal and on the Canary Islands in 2011–2014 (KT878660, 99.74% identity; KT878661, 99.73%; KT878657, 99.75%; KC572861, 99.75%; KT878656, 99.75%; KJ139454, 100%; KT878658, 99.52%; KP835987, 99.80%; and KP836003, 99.74%; Supplementary Table S2). In contrast, the p-distance values for the P gene sequences of other Spanish Mediterranean DMV outbreaks were higher. Interestingly, the p-value was lower for the sequences from the 1990 outbreak (p ≈ 0.012, AJ608288) than for those of the 2007 and 2011 outbreaks (p ≈ 0.020, EU039963, HQ829973, JN210891). The p-distance value with the P gene sequence (KU977450) from a fin whale that stranded in Italy in 2013 was also relatively high (0.019, Supplementary Table S2).

The N gene sequence also exhibited shorter phylogenetic p-distances with the isolate of year 2012 from the Spanish Mediterranean (MG000863) and with some Atlantic-found sequences (Supplementary Table S3). Thus, the estimated p-distances were 0.005 for the 2012 Spanish sequence and for a German North Sea isolate (EF469546), and 0.016 for some Atlantic sequences (KP835984). Higher p-distances (values of 0.021) were found for the isolates from the Mediterranean outbreaks of 1990 (AJ608288), 2007 (HQ829973) and 2011 (MG773792), and for the isolates from a fin whale (KU977449) and a Cuvier’s beaked whale (KX237510) that stranded in Italy in 2013 and 2015, respectively.

## Discussion

The results of the present analyses revealed DMV infection in seven of the eight stranded striped dolphins examined herein. In the eighth dolphin, a well-preserved specimen (animal 4) stranded during the same period, the histological and virological assays were negative for DMV. In four of the eight stranded animals in which a histological analysis was possible, this analysis evidenced variable degrees of non-suppurative meningoencephalitis. The CDV immunopositivity of these animals, and the positivity in all the animals except for animal 4, of the molecular tests that targeted the CeMV P, N and Fusion (F) genes, evidenced DMV infection in seven of the eight stranded animals. As previously observed^33^, the high-sensitivity rt PCR and the RT-PCR assays also allowed the detection of viral RNA in decomposed tissues. The PCR positivity in all the tissues of these seven positive animals was more consistent with systemic DMV infection than with the central nervous system-restricted chronic encephalitic form^34^ that has been reported since 2011 in several animals stranded on the Italian Mediterranean coast^18–20^. So, even though most of the DMV infection cases that occur outside outbreak periods appear to take the chronic encephalitic form^25,34^, our data suggest the occurrence of possible systemic infection cases during inter-epizootic periods.

The identity of the partial P and N viral sequences indicates that all positives were infected by the same DMV strain, a strain for which sequence comparisons revealed substantial differences with the DMVs found in the previous Mediterranean outbreaks of 1990^2^, 2007^15^ and 2011^17^ (Fig. 3), while showing a striking similarity to the sequences of the DMVs circulating in cetaceans in the European Atlantic and Northern seas; e.g., the DMV strains found in 2007 in a white-beaked dolphin on the German Coast^31^, in 2011 in a striped dolphin from the Canary Islands^28^, and in the viruses detected in 2012 and 2013 on the northwestern coast of the Iberian Peninsula^29^. The molecular data strongly suggest a link between these Atlantic DMV strains and those described in the present study, found in dolphins stranded on Sicilian coasts. Moreover, similarities to the sequences of the DMVs from the five striped dolphins stranded in 2012–2016 on the Spanish Mediterranean coastline^25^ suggest the recent spread in the western Mediterranean of this DMV strain of Atlantic origin which appears to have already been detected in Germany in 2007^31^. Indeed, while viral sequences from the Mediterranean outbreaks of 1990, 2007 and 2011 have suggested that these outbreaks were caused by closely related DMV strains^17,32^, the partial P and N gene sequences described herein cluster separately from these earlier outbreak DMV sequences (Z47758, HQ829973, JN210891 in Fig. 3).

The spreading of the present strain from a strain found in the Atlantic as far back as 2007 was first detected in the Mediterranean Basin in 2012, specifically in the Western Mediterranean. This spreading reinforces pre-existing evidence for a close relationship between the DMV strains circulating in the Mediterranean Sea and the Atlantic Ocean^9,15,28^, perhaps in relation to both the social behaviour of cetacean species and the Gibraltar Strait connection between the Mediterranean Sea and the Atlantic Ocean^9,15,35–37^. Nevertheless, the closeness between the present DMV strain and that found in 2012 in the Spanish Mediterranean would also agree with the view that the present one has been circulating among Mediterranean striped dolphins since 2012. Obviously, the possibility^4,17^ of endemic DMV circulation occurring in the Mediterranean should consider the transmission and maintenance of DMV strains among not only striped dolphins, but also other cetaceans, because pilot whales and a Cuvier’s beaked whale were infected with DMV^22,24,32^. In fact, the association between the different cetacean species living in the Mediterranean Sea could be a critical factor to ensure virus persistence in this sea^9,38,39^. However, the low density of whales and beaked whale species in the Mediterranean (<0.012 beaked whales/nautical mile; 3% of the total cetaceans population)^40^ suggests that the involvement of these cetaceans as DMV reservoirs is much less likely than with the much more abundant striped dolphins (0.46 individuals per nautical mile; 95% of the cetaceans in the Mediterranean)^40^.

The RT-PCRs^41,42^ and rt PCR^43^ assays used herein confirmed the CeMV diagnosis, and the sequence analysis of the amplicons from the RT-PCR assays supplied useful data to help understand the molecular epidemiology of DMV. As with most sequences available in the GenBank for CeMVs, ours are partial gene sequences of various lengths. Future studies on longer sequences or complete CeMV genomes are anticipated to further resolve how DMV strains evolve and circulate. Additional studies should also address the question of whether the non-synonymous substitutions observed in the P gene among different strains (Table 3) are biologically relevant, which falls in line with previously made proposals in a former Italian study^26^. In addition to P and N gene sequence analyses, further contributions could be made by including in the sequence analysis the viral haemagglutinin (H) gene, which has been shown to be highly variable among CDV strains^44,45^, particularly since the sequence variations in this gene may have consequences on host range, virulence and neutralising epitopes^46^. Previously published RT-PCR protocols^23,32,47^ could be used in the future to obtain DMV H gene sequences, which could help to understand the molecular epidemiology and the virulence determinants of DMV strains. All this, together with P and N gene sequences, could provide useful markers for tracking these viruses in space and time terms.

In short, our results suggest that the closely related DMV strains of a remote Atlantic origin are circulating and possibly causing systemic infection in striped dolphins in the eastern Mediterranean. The P and N gene sequences differ more from the previously collected Mediterranean DMV sequences than from the recently reported sequences in the dolphins that have stranded on the Spanish Mediterranean coastline since 2012. These data indicate that DMV strains of an Atlantic origin are circulating in the Mediterranean and affect striped dolphins. They stress the need for further research to determine not only how this DMV strain circulates, but also its potential impacts on the cetacean populations living in the Mediterranean Sea.

## Methods

Between August and October 2016, eight striped dolphins were stranded and found dead on the SE coastline of Sicily (Italy) encompassing the Mediterranean and Ionian Seas (Fig. 1). Within 24 hours after stranding, these animals were subjected to post-mortem examinations at the Istituto Zooprofilattico Sperimentale (IZS) della Sicilia “A.Mirri” (Palermo, Italy) following previously published protocols^27^. The necropsy of each dolphin was performed separately, with exhaustive disinfection measures taken at the site and of the material after each necropsy. The level of carcass preservation at the time of necropsy was graded according to the code 1 to 5 system^27^. The gender and details of each stranded dolphin are provided in Table 1.

Fresh tissue samples from brain, cerebellum, lung, pulmonary lymph nodes, heart, spleen, liver, intestine, kidneys and urinary bladder, as well as swabs of blowhole, were collected for the histopathological and molecular diagnostics. Samples for histopathological analysis were fixed in 10% neutral buffered formalin and processed according to standard procedures for haematoxylin-eosin staining. Immunoperoxidase histochemistry (IHC) was performed on the selected formalin-fixed paraffin-embedded (FFPE) brain section with an IgG2B-isotype monoclonal anti-NP antibody for the nucleoprotein of CDV (CDV-NP; LifeSpan BioSciences®, Inc., USA)^17,48^, known to cross-react with CeMV. Positive controls were similar sections from a CDV-positive canine brain. Background staining was assessed by replacing the primary antibody with bovine serum albumin^48^.

For the molecular diagnostics, samples were kept at -20 °C and analysed under a quality assurance system to also prevent cross-contamination. The collected samples were tested by PCR for CeMV, adenovirus and herpesvirus. For this purpose, frozen tissues were thawed and homogenised (10% w/v) as previously reported^49^, storing the supernatant at -80 °C until assayed.

RNA and DNA were extracted from the homogenates by respectively using the QIAamp^®^ Viral RNA Mini and DNeasy Blood & Tissue Kits (Qiagen, Hilden, Germany), according to the manufacturer’s instructions. The DNA from liver, kidney, lung and intestine were PCR-amplified to detect adenoviruses^50^ and herpesviruses^51^, as previously reported. For these purposes, GoTaq^®^ G2 DNA Polymerase (Promega Italia s.r.l., Milan, Italy) and the Taq PCR Core Kit (Qiagen, Hilden, Germany) were respectively used. For CeMV detection, the extracted RNA was subjected to a previously described^43^ rt PCR assay that uses probes of the Universal Probe Library (UPL) targeting the F gene of DMV, employing for amplification the QuantiTect^®^ Multiplex RT-PCR Kit (Qiagen, Hilden, Germany). This rt PCR was firstly carried out on the tissue samples routinely used to detect CeMV (brain, kidney, lung, pulmonary lymph nodes, spleen, intestine), and subsequently on the other collected tissue samples (cerebellum, liver, urinary bladder, blowhole swab). The negative and positive controls for these rt PCR assays were, respectively, nuclease-free water and previously extracted RNA samples from a lung homogenate of a CeMV-positive dolphin (from the 2011 outbreak in the Spanish Mediterranean)^17^.

The RNA from the different samples of each dolphin (animals 1: brain and lung; animal 2, 7 and 8: lung and pulmonary lymph node; animal 3: spleen and pulmonary lymph node; animal 5: lung and spleen; animal 6: lung and urinary bladder) were subjected to conventional RT-PCR assays, run to target a fragment of the P and N genes, in line with published protocols^41,42^, using the QIAGEN^®^ OneStep RT-PCR Kit **(**Qiagen, Hilden, Germany). In all the RT-PCR assays, RNA that was previously extracted from a canine distemper virus (CDV) strain (strain Bussell) served as a positive control, and nuclease-free water as negative control. For sequencing purposes, the P and N gene fragments amplified from each tissue and each animal were purified with the Illustra^TM^ GFX^TM^ PCR DNA and Gel Band Purification Kit (GE Healthcare Life Sciences, Amersham, Buckinghamshire, UK), and were submitted to a Sanger sequencing service (ABI Prism 3730 automated sequencer from Applied Biosystems, Foster City, CA, USA).

The obtained partial P and N gene sequences were subjected to BLASTN analyses (http://blast.ncbi.nlm.nih.gov) to search for related CeMV sequences in GenBank. The nucleotide and inferred amino acid (aa) sequences were aligned, compared with the retrieved sequence data and analysed by the BioEdit ver. 7.2.5 software^52^.

The phylogenetic analyses were carried out using the MEGA X software^53^. P-distance matrices were calculated and tree topology was inferred by Maximum Likelihood based on p-distances (bootstrap on 1,000 replicates, generated with a random seed). These sequence data, together with the partial N gene sequences from the striped dolphins recently stranded in Spain^25^, have been submitted to the GenBank/EMBL/DDBJ databases with the following accession numbers: P gene (MG000861) and N gene (MG000862) from the Sicilian strandings; N gene (MG000863) from the Spanish strandings.

### Accession codes

The sequence data of this manuscript have been submitted to the GenBank/EMBL/DDBJ databases with the following accession numbers: MG000861, MG000862, MG000863.

## Supplementary information

Supplementary Tables

## Data Availability

Sequences have been submitted to the GenBank/EMBL/DDBJ databases with the following accession numbers: MG000861, MG000862 and MG000863.
