# Supplementary material for: Circulation of a novel strain of dolphin morbillivirus (DMV) in stranded cetaceans in the Mediterranean Sea
Source: Sci Rep. 2019 Jul 5;9:9792. doi: 10.1038/s41598-019-46096-w (PMC6611785; doi:10.1038/s41598-019-46096-w)
Supplement: Supplementary file 1 — Supplementary Tables [file 41598_2019_46096_MOESM1_ESM.pdf]

# Circulation of a novel strain of dolphin morbillivirus (DMV) in stranded cetaceans in the Mediterranean Sea

**Francesco Mira[1*+], Consuelo Rubio-Guerri[2,3+], Giuseppa Purpari[1+], Roberto Puleio[1], Giulia Caracappa[1], Francesca Gucciardi[1], Laura Russotto[1], Guido Ruggero Loria[1], and Annalisa Guercio[1]**

[1]Istituto Zooprofilattico Sperimentale della Sicilia "A. Mirri", Palermo, 90129, Italy

[2]Fundaciòn Oceanografic de la Comunitat Valenciana, Valencia, 46013, Spain

[3]VISAVET-Animal Health Department, Veterinary School, Complutense University, Madrid, 28040, Spain

[*]dottoremira@gmail.com

[+]These authors contributed equally to this work

| | CODE* | MACROSCOPIC LESIONS | | | MICROSCOPIC LESIONS | | |
|---|---|---|---|---|---|---|---|
| | | **Pulmonary atelectasis** | **Enlarged lymph nodes** | **Congested brain** | **Evidence of diffuse interstitial pneumonia** | **Lymph nodes with lymphocyte depletion and lymphocytolysis** | **Microscopic evidence of non-suppurative meningoencephalitis** |
| **Animal 1** | 2 | — | — | X | — | X | X |
| **Animal 2** | 2 | — | — | — | — | — | X |
| **Animal 3** | 3 | — | — | — | — | — | X |
| **Animal 4** | 2 | — | — | — | — | — | — |
| **Animal 5** | 4 | Autolysed carcass | | | NT | NT | NT |
| **Animal 6** | 3 | — | — | — | — | — | X |
| **Animal 7** | 2 | — | — | — | — | — | X |
| **Animal 8** | 3 | — | — | — | — | — | X |

**Supplementary Table S1: Summary of main macroscopic and microscopic lesions found in the eight animals.** * level of carcass graded according to the code 1 to 5 system. NT: Samples not tested.

| ID/GeneBank acc number | p-distance with MG000861 | %identity with MG000861 |
|---|---|---|
| PWMV-AF200817-Gm/2000/At | 0.107 | 89.68 |
| PWMV-FJ842381-Gmac/1996/At | 0.138 | 86.87 |
| PWMV-KT006289-Gmac/2015/At | 0.122 | 88.33 |
| PWMV-KT006289-Gma/2015/At | 0.122 | 88.33 |
| PWMV-KT006290-Gma/2015/At | 0.122 | 88.30 |
| DMV-KJ139451-Sc/2002/At | 0.037 | 98.64 |
| DMV-KT878660-Sc/2009/At | 0.003 | 99.74 |
| DMV-KT878659-Sc/2014/At | 0.007 | 97,88 |
| DMV-KT878661-Sc/2012/At | 0.003 | 99,73 |
| DMV-KT878657-Sc/2012/At | 0.003 | 99.75 |
| DMV-KC572861-Sc/2012/Me | 0.003 | 99.75 |
| DMV-KT878656-Sc/2013/PorAt | 0.003 | 99.75 |
| DMV-KJ139454-Sc/2011/At | 0.000 | 100 |
| DMV-KT878658-Sc/2012/At | 0.005 | 99,52 |
| DMV-KF695110-Sc/2009/At | 0.012 | 98.78 |
| DMV-KJ139453-Tt/2005/At | 0.017 | 98.36 |
| DMV-KJ139452-Sc/2007/At | 0.018 | 98.28 |
| DMV-KX237511 _Zc/2015/Me | 0,024 | 97.20 |
| DMV-EU039963-Gm/2007/Me | 0,020 | 97.74 |
| DMV-KU977450-Bp/2013/Me | 0,014 | 98.35 |
| DMV- KR337460-Bp/2011/Me | 0.017 | 98.35 |
| DMV-JN210891-Sc/2011/Me | 0,020 | 98.20 |
| DMV-KP835987-Sc/2012/At | 0,002 | 99.75 |
| DMV-KR704575-Ip/2013/Pa | 0.017 | 98.34 |
| DMV-HQ829972-Gm/2007/Me | 0.024 | 97.43 |
| DMV-Z47758-Sc/1990/Me | 0.021 | 97.67 |
| DMV-AJ608288-Sc/1990/Me | 0.012 | 97.66 |
| DMV-KC888945-La/2011/No | 0.028 | 97.26 |
| DMV-KC888946-La/2011/No | 0.027 | 97.33 |
| PMV-KF650727-Pp/1990/No | 0.083 | 92.30 |
| DMV-KP836003-Dd/2013/At | 0.002 | 99,74 |
| DMV-KY681807-Bp/2016/No | 0.018 | 98.60 |

**Supplementary Table S2: P-distances and % of identity of our sequence (MG000861) with the sequences that are in Fig 2 (phylogenetic tree) for phosphoprotein (P) gene.** The name of each sequence is composed of the virus strain name (DMV, dolphin morbillivirus; PMV, porpoise morbillivirus; PWMV, pilot whale morbillivirus), GenBank accession number, cetacean species host (Sc, *Stenella coeruleoalba*; Gm, *Globicephala melas*; Gma, *Globicephala macrorhynchus;* Tt, *Tursiops truncatus*; Zc, *Ziphius cavirostris*; Bp, *Balaenoptera physalus*; Ip, *Indopacetus pacificus*; La, *Lagenorhynchus albirostris;* Pp, *Phocoena phocoena*, Dd, *Delphinus delphis*), year and geographic area of stranding (At, Atlantic Ocean; Me, Mediterranean Sea; No, North Sea; Pa, Pacific Ocean).

| ID/GeneBank acc number | p-distance with MG000862 | %identity with MG000862 |
|---|---|---|
| DMV-HQ829973-Sc/2007/Me | 0.021 | 97.91 |
| DMV-HQ829972-Gm/2007/Me | 0.021 | 97.91 |
| PMV-AY949833-Pp/2005/No | 0.122 | 88.12 |
| PWMV-AF200818-Gm/2000/At | 0.146 | 85.41 |
| DMV-AJ608288-Sc/1990/Me | 0.021 | 97.91 |
| DMV-KX237510-Zc/2015/Me | 0.021 | 97.91 |
| DMV-KU977449-Bp/2013/Me | 0.021 | 97.91 |
| CeMV-KP836004-Dd/2013/At | 0.011 | 98.95 |
| CeMV-KP836000-Dd/2012/At | 0.011 | 98.95 |
| CeMV-KP835992-Sc/2012/At | 0.011 | 98.95 |
| CeMV-KP835988-Sc/2012/At | 0.016 | 98.43 |
| CeMV-KP835984-Sc/2011/At | 0.016 | 97.91 |
| DMV-MG000863-Sc/2012/Me | 0.005 | 99.5 |
| CeMV-KT878655-Sc/2014/At | 0.027 | 97.39 |
| CeMV-KT878654-Sc/2012/At | 0.027 | 97.39 |
| CeMV-KT878653-Sc/2013/At | 0.021 | 97.91 |
| CeMV-KP835998-Sc/2007/At | 0.021 | 97.39 |
| DMV-EF469546-La/2007/No | 0.005 | 99.47 |

**Supplementary Table S3: P-distances and % of identity of our sequence (MG000861) with the sequences that are in Fig 2 (phylogenetic tree) for nucleoprotein (N) gene.** The name of each sequence is composed of the virus strain name (DMV, dolphin morbillivirus; PMV, porpoise morbillivirus; PWMV, pilot whale morbillivirus), GenBank accession number, cetacean species host (Sc, *Stenella coeruleoalba*; Gm, *Globicephala melas*; Gma, *Globicephala macrorhynchus;* Tt, *Tursiops truncatus*; Zc, *Ziphius cavirostris*; Bp, *Balaenoptera physalus*; Ip, *Indopacetus pacificus*; La, *Lagenorhynchus albirostris;* Pp, *Phocoena phocoena*, Dd, *Delphinus delphis*), year and geographic area of stranding (At, Atlantic Ocean; Me, Mediterranean Sea; No, North Sea; Pa, Pacific Ocean.
